# Post-embryonic Development of the Circadian Clock Seems to Correlate With Social Life Style in Bees

**DOI:** 10.3389/fcell.2020.581323

**Published:** 2020-11-12

**Authors:** Katharina Beer, Charlotte Helfrich-Förster

**Affiliations:** Department of Neurobiology and Genetics, Biocenter, University of Würzburg, Würzburg, Germany

**Keywords:** social, honey bee, solitary bee, circadian clock, activity rhythm, neuronal network, development

## Abstract

Social life style can influence many aspects of an animal’s daily life, but it has not yet been clarified, whether development of the circadian clock in social and solitary living bees differs. In a comparative study, with the social honey bee, *Apis mellifera*, and the solitary mason bee, *Osmia bicornis*, we now found indications for a differentially timed clock development in social and solitary bees. Newly emerged solitary bees showed rhythmic locomotion right away and the number of neurons in the brain that produce the clock component pigment-dispersing factor (PDF) did not change during aging of the adult solitary bee. Honey bees on the other hand, showed no circadian locomotion directly after emergence and the neuronal clock network continued to grow after emergence. Social bees appear to emerge at an early developmental stage at which the circadian clock is still immature, but bees are already able to fulfill in-hive tasks.

## Introduction

Social interactions are known to influence behavioral rhythms in different animals (Sharma et al., 2004; Favreau et al., 2009; Eban-Rothschild and Bloch, 2012). Nevertheless, it is largely unexplored how social life style shapes the circadian system of animals.

The European honey bee, *Apis mellifera*, is an important model organism to study the impact of sociality on the circadian clock (Bloch, 2010; Eban-Rothschild and Bloch, 2012; Beer and Bloch, 2020). The honey bee displays the highest form of sociality known in insects, called eusociality. It is defined by (1) cooperative brood care (2) overlapping generations in one colony, and (3) reproductive division of labor with several sterile workers and one or a few fecund colony members (Winston, 1987; Michener, 2000). Honey bee colonies usually consist of one queen that reproduces with the male bees (drones) and several thousand worker bees, which take care of the colony. They do this in an age dependent manner, meaning that younger bees take care of the brood and older bees perform tasks like attending the queen, nest building and guarding (Free, 1965; Seeley, 1982; Robinson et al., 1989, 2005). The last stage in their life is becoming a forager bee.

The circadian clock plays an essential role for honey bee survival: forager bees show daily rhythms in flight times and a remarkable time memory, allowing them to synchronize with the most rewarding flowering times of the day (Kleber, 1935; Moore, 2001). Furthermore, the bees need their circadian clock for sun compass orientation (Lindauer, 1960).

The molecular basis of the circadian clock consists of transcriptional/translational feedback loops in the brain that drive circadian rhythms in behavior and physiology (Dunlap, 1999; Bloch, 2010). One of the major neuropeptides that controls rhythmic behavior in insects is pigment-dispersing factor (PDF) (Shafer and Yao, 2014; Beer et al., 2018). The neurons expressing PDF are part of the honey bee clock and build a complex network that connects the clock with brain centers controlling locomotion, complex behaviors and the endocrine system (Fuchikawa et al., 2017; Beer et al., 2018).

As in other animals, the circadian clock of the honey bee “free-runs” with an endogenous period of circa 24 h in the absence of environmental time cues (Zeitgebers) (Edery, 2000). It is synchronized to the 24-h day by environmental light and temperature cycles and by social cues from the colony (Frisch and Koeniger, 1994). Social cues can even dominate the other Zeitgebers, demonstrating the profound impact of social life style on the honey bee’s circadian clock (Beer et al., 2016; Fuchikawa et al., 2016). Social cues from the colony are furthermore beneficial for clock development in individual bees (Eban-Rothschild et al., 2012; Beer et al., 2016). Young bees that perform nursing tasks are arrhythmic and active around the clock, a behavior, which is believed to support rapid colony development (Moore et al., 1998; Bloch and Robinson, 2001; Bloch et al., 2013). Signals from the colony appear to help these nurse bees to mature and become rhythmic foragers: young bees kept isolated from the colony take longer to develop behavioral circadian rhythms than bees that have been in social contact (Eban-Rothschild et al., 2012; Beer et al., 2016). Most importantly, the circadian clock and the honey bee’s behavior is highly plastic and strongly influenced by social cues, because foraging bees can revert to nursing without rhythms and young bees can start foraging prematurely with daily activity rhythms (Bloch and Robinson, 2001). All this depends on the needs of the colony.

In contrast to the honey bee, most other bee species pursue a solitary life style (Michener, 2000). The red mason bee, *Osmia bicornis*, is a generalist pollinator native to Europe, which lately has received increasing attention as a highly promising species for pollination services (Garibaldi et al., 2013; Sedivy and Dorn, 2014). As solitary bees, they do not show reproductive labor division and the young bees are not born in the safety of a bee hive, but emerge in spring from small nests, in which they overwintered in diapause (Raw, 1972). They have to cope with daily environmental changes right after emergence, which requires a fully matured circadian system. In our study, we hypothesized that the honey bee takes advantage of the social colony environment and emerges with an immature circadian clock compared to the clock system of the solitary bee. If this were to be the case, we expected that:

(I) Solitary bees display circadian behavior right after emergence, but honey bees do not.
(II) The clock network in the brain of honey bees shows continued maturation after the bee’s emergence in contrast to the clock network in solitary bees.

## Materials and Methods

Honey bees: Honey bee colonies, species *A*. *mellifera*, subspecies *carnica*, were kept at the University of Würzburg, Department of Animal Ecology and Tropical Biology. Queens were inseminated by multiple drones. Standard beekeeping methods were applied with bees kept in field colonies consisting of approximately 35,000–40,000 bees.

Solitary bees: Cocoons of solitary bees, species *O*. *bicornis*, were purchased at a commercial beekeeper (WAB-Mauerbienenzucht, Konstanz) in autumn (2014, 2015, and 2016) and stored at 4°C and 60% temperature and humidity (RH) until the start of the experiments in the following spring.

We tested our hypothesis of an immature circadian system in the honey bee via a behavioral assay of monitoring locomotion after emergence of the social honey bees and the solitary mason bees. Furthermore, insect brain tissues of different developmental stages in the bees were stained via immunofluorescence to visualize the development of the clock neuronal network.

### Circadian Rhythm Onset in Bee Behavior

In a set of behavior experiments, we assessed if the ontogeny of circadian rhythms in activity during post-emergence development of *O. bicornis* differs from the one in *A. mellifera*.

#### Monitoring Locomotion

Brood combs of *A. mellifera* colonies were kept overnight in a climate chamber [constant darkness (DD), 20°C, 60% RH, DD] and newly emerged female worker honey bees were collected for the behavioral experiments under dim red light. We introduced newly emerged bees into an infrared (IR)-beam based activity monitoring system (LAM-system, TriKinetics Inc., Waltham, MA United States) and monitored their locomotion under constant conditions (DD and RH; for details see Table 1). In an additional treatment, we monitored locomotion of newly emerged honey bees that were separated by a double mesh wire from a miniature bee colony next to the monitoring system. The miniature colony consisted of a brood comb (open and closed brood) with approximately 1,000 bees (hive bees and foragers) on it. Queen pheromone (Bee Boost with queen mandibular pheromone) substituted a real queen to reduce stress caused by removing the bees from the hive and their queen. We added this treatment, because the social contact to the colony increases survival of the honey bees in the set up (Beer et al., 2016). In three further trials, we introduced cocoons of *O. bicornis* into the monitoring tubes and monitored their locomotion after their emergence under constant environmental conditions (DD and RH; see Table 1). We monitored bee activity for as long as they lived in the set up, which was 3–45 days. We performed our experiments at temperatures, which young honey bees and solitary bees may experience in nature (Fahrenholz et al., 1989; Strohm et al., 2002). We decided to submit the honey bees to 30°C, which is a bit lower than the core hive temperature, because there were also forager bees in the miniature colony present that are typically not found in the hive center (Seeley, 1982; Klein et al., 2014). *O. bicornis* bees seem to favor temperatures of 20°C (or above) for emergence (Beer et al., 2019). Therefore, we performed the solitary bee experiments at approximately 20 and 25°C. We chose two different temperatures, because the mason bee is submitted to stronger temperature fluctuations than the honey bee in the thermoregulated hive, but we avoided extreme temperature conditions. The bees (honey bees and solitary bees) have never been exposed to light or oscillating temperature.

**TABLE 1:** Rhythms in locomotor activity of newly emerged individuals of social (*A. mellifera*) and solitary (*O. bicornis*) bees.

	*Apis 1*	*Apis 2*	*Osmia 1*	*Osmia 2*	*Osmia 3*
Environmental conditions	30°C, 60% RH	30°C, 60% RH	20°C, 60% RH	19.2°C, 60% RH	25°C, 45% RH
*N* (male)	–	–	8	10	13
Rhythmic males	–	–	8	8	12
Percent of rhythmic males	–	–	100%	80%	92%
*N* (female)	15	25	9	40	7
Rhythmic females	0	0	9	26	7
Percent of rhythmic females	0%	0%	100%	65%	100%
Percent of rhythmic individuals	0%	0%	100%	68%	95%
*p*-value	<0.001	<0.001	<0.001	<0.05	<0.001

*Environmental conditions, sample size, and absolute number as well as percentage of rhythmic individuals of five different experiments (*Apis 1* and *2* with *A. mellifera* and *Osmia 1*–*3* with *O. bicornis*). We monitored the activity of bees in experiments *Apis 1* and *Osmia 1*–*3* individually without any contact to other bees. In experiment *Apis 2* locomotor activity of individual bees was monitored, which had social contact to a mini honey bee colony. In experiment *Osmia 3* monitor tubes with two different diameters (16 and 25 mm) were used. There are significantly more arrhythmic individuals in the honey bee experiments and significantly more rhythmic individuals in the *Osmia* experiments (*p*-values and binomial test).*

#### Data Analysis

The activity data was registered in counts (beam crosses) per minute in the TriKinetics system and evaluated with ActogramJ software plugin for ImageJ (Schmid et al., 2011) (Fiji ImageJ Version 1.49, Wayne Rasband, National Institutes of Health, Bethesda, MD, United States). We evaluated rhythmicity in locomotion only for bees that showed activity for at least three consecutive days in the analysis. Circadian rhythmicity during the first 2 days after emergence of the bees was determined by eye (detection of a circadian pattern of activity in the actogram; e.g., re-occurring activity at more or less the same time for consecutive days), because available statistical tests for periodicity are often not precise enough when only a few days are evaluated (Refinetti et al., 2007). Statistical analysis was conducted in R (R version 3.2.2) using R stats package.

### Immunocytochemistry in the Clock Network of Bees

Honey bees were sampled after they were raised by the colony. For this, we reintroduced newly emerged honey bees (overnight in an incubator at 35°C and 60% RH) into the colony after marking them with paint on the thorax and sampled the marked honey bees at specific ages. Solitary bees were submitted to daily cycles of fluctuating temperature and light conditions (12 h of 25°C and light and 12 h of 15°C and darkness) and kept all together until sampling in a big flight tent (size: 60 cm × 60 cm × 56 cm) that was equipped with sugar syrup (APIinvert^®^, Südzucker, Mannheim, Germany), pollen, and water *ad libitum* as well as tubes for providing hiding places and clay. We performed sampling in all groups of bees always at the same time of the day, which resembled the subjective morning (Zeitgeber time 0–1.5).

#### Staining Clock Neurons in Bee Brains

Bees were demobilized on ice, decapitated and a window was cut into the cuticle of the frontal head. Heads were then fixed in 4% PFA in PBST (0.1% Triton) for 3–4 h on a shaker. All washing and incubation steps were performed on a shaker at lowest speed setting. After washing the heads in PBS three times (10 min each), brains were dissected in PBS and washed again three times in PBS. Samples were stored in 100% methanol until all differently aged bees were sampled (step wise dehydration and rehydration in methanol/PBS solution: 0, 30, 50, 70, 90, and 100%, each 10 min). We added an additional fixation step with Zamboni’s fixative over night at 4°C to reduce the background staining in the whole mount brains. Brains were washed three times in PBS and incubated in sodium citrate buffer (10 mM, pH 8.5) at 80°C in an antigen retrieval step. Afterward we washed the brains 10 times in PBS, two times in PBST (0.5%), one time in PBST (2%) and one time in PBST (0.5%) and pre-incubated in 5% NGS-PBST (0.5%) at 4°C overnight. The brains were then incubated in 1:3,000 anti-β-Pigment Dispersing Hormone (PDH) antibody solution [in 5% NGS-PBST (0.5%)] and 0.02% NaN_3_ for 6 days at 4°C and 1 day at RT. PDH antibody recognizes the PDF peptide in various insects, including hymenoptera (Bloch et al., 2003; Weiss et al., 2009; Fuchikawa et al., 2017; Beer et al., 2018; Kay et al., 2018). The brains were washed six times in PBST (0.5%), incubated in the secondary antibody solution [1:200 Alexa Flour 635 goat anti rabbit in 5% NGS-PBST (0.5%)] and then washed again [three times in PBST (0.5%) and three times in PBS], before we mounted them in mounting medium for fluorescence (Vectashield, Vector laboratories, Burlingame, CA, United States). We mounted the brains between two microscope cover glasses with spacers in mounting medium for fluorescence, which prevented the tissue from being crushed.

#### Data Analysis

Immunocytochemistry data was analyzed with a Leica TCS SPE confocal microscope (Leica microsystems, Wetzlar, Germany) equipped with 10×/0.30 CS ACS APO and 20×/0.60 IMM CORR ACS APO objectives. Brains were scanned sequentially in stacks (Leica Application Suite Advanced Fluorescence 2.7.3.9723, Leica Microsystems, Mannheim, Germany). Samples of one experiment were processed with the same settings and obtained confocal pictures (resolution 1024 × 1024) were further processed in Fiji ImageJ (Version 1.49, Wayne Rasband, National Institutes of Health, Bethesda, MD, United States). Quantification of cell number was done by counting stained cells in both hemispheres and then averaging for the brain. Subsequently, brightness and contrast were adjusted in the figures displayed in the results section. Statistical analysis was conducted in R (R version 3.2.2) using R stats package.

## Results

### Circadian Rhythms in Locomotion of Newly Emerged Bees

**FIGURE 1:**
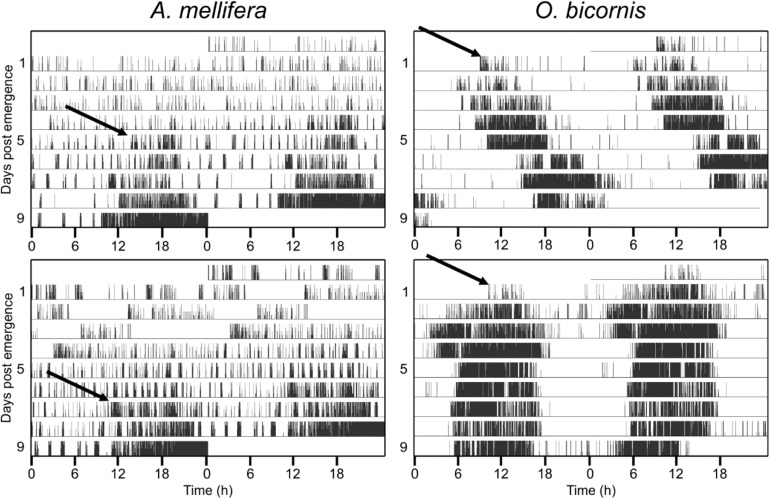
Ontogeny of circadian activity rhythms in social (*A. mellifera*) and solitary (*O. bicornis*) bees. Four example actograms for the monitored activity in *A. mellifera* and *O. bicornis*. Actograms are double plotted, showing two consecutive days in a row, in order to better visualize the circadian pattern in activity rhythms. The honey bee *A. mellifera* never shows circadian activity during the first few days. In the example actograms, the honey bees start to exhibit circadian rhythmicity from the fifth day (upper panel) and seventh day (lower panel) onward (arrows). The solitary bees (*O. bicornis*) show circadian activity right after emergence from their cocoon (arrows in the right actograms).

None of the honey bees in our experiments exhibited circadian rhythms during the first 2 days after their emergence (Figure 1). On the contrary, most of the solitary *Osmia* bees (mean for all experiments ± STE: 88% ± 0.1) showed circadian rhythmicity in their activity right after their emergence and this was the case under different temperature conditions and for both sexes (Exact binomial test and *p* < 0.05) (Figure 1 and Table 1). The few individuals that did not show strong rhythmicity died only a few days after their emergence.

### PDF Network Staining in Bees of Different Ages

**FIGURE 2:**
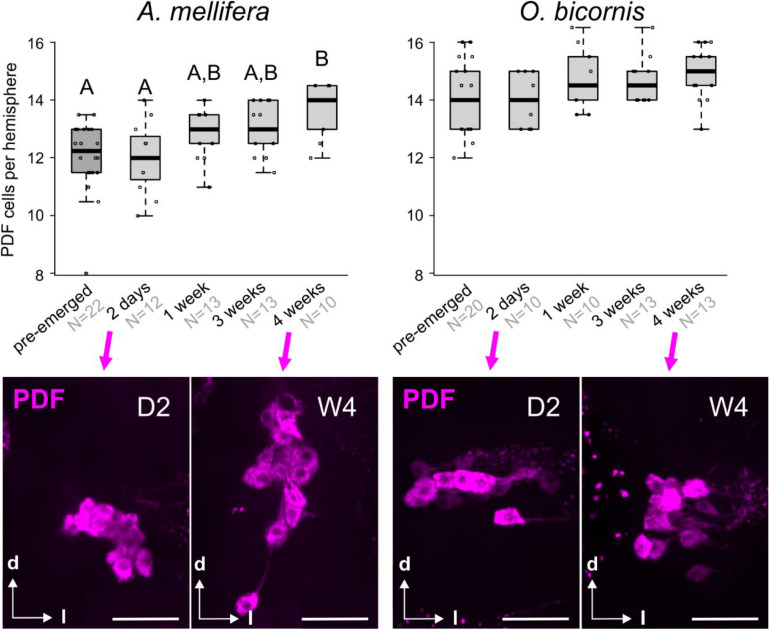
Post-emergence development of the clock neuronal network in social bees (*A. mellifera*) and solitary bees (*O. bicornis*). Top row: The graphs display the number of PDF cells per brain hemisphere (boxplots with data points) in differently aged *A. mellifera* (left) and *O. bicornis* (right) bees. “Pre-emerged” means bees were sampled approximately 2–3 days before the bees would have emerged. In honey bees, pupal stages P7 and P9 are equivalents to pre-emerged *O. bicornis* bees, because these would have also emerged 2–3 days after sampling (the cell numbers of pre-emerged *A. mellifera* are calculated from a different unpublished experiment). Different letters indicate statistical difference in cell number. Sample sizes are indicated in light gray with the sample group. Second row: example pictures of PDF cell clusters (magenta) in *A. mellifera* (left) and *O. bicornis* (right) bees for the ages of 2 days (D2) and 4 weeks (W4). Scale bar: 50 μm.

The PDF-immunostaining of pre- and post-emerged bees of different ages showed that, in honey bees, the number of PDF neurons steadily increased with age (Kruskal Wallis rank sum test: 19.3, *df* = 4, *p* < 0.01; *post-hoc* test: pairwise comparison using Wilcoxon rank sum test) (Figure 2, left graph). In contrast, we could not detect such increment in *Osmia*, and the PDF cell number was approximately 14–15 neurons per hemisphere for all ages (Figure 2, right panels). Although we could see a slight increase in the PDF cell number from 2-day old mason bees to older mason bees (Figure 2, right graph) this turned out to be not significant (Kruskal Wallis rank sum test; *post-hoc* test: pairwise comparison using Wilcoxon rank sum test). Interestingly, in the two-day-old honey bee brains, the PDF cell bodies cluster closer together than it is the case in the older honey bees and *Osmia* of all ages (Figure 2, second row).

## Discussion

Our study indicates that the circadian clock system in newly emerged honey bees is immature at two levels: (I) behavioral output of the bee clock and (II) anatomy of the brain clock. While the neuronal honey bee clock network continues to grow moderately, but significantly after emergence (either due to continued cytogenesis or expression of PDF in more neurons in aged bees), the number of clock cells expressing PDF in solitary bees does not increase after the time point of emergence. This indicates that the circadian system of the solitary bees was mature at emergence, while that of the honey bees was immature. Our behavioral experiments confirm this assumption, because young solitary bees show rhythmic behavior right after emergence, while young honey bees remain behaviorally arrhythmic for some days.

### Timing of Circadian Onset of Behavioral Rhythms Is Delayed in Adult Honey Bees

Several earlier studies suggested an important role of the social lifestyle of honey bees in circadian behavior and the normal development of the circadian system (Bloch and Robinson, 2001; Eban-Rothschild et al., 2012; Beer et al., 2016; Fuchikawa et al., 2016). Several studies discovered that the social environment plays an important role in the ontogenetic process of circadian behavior and bees kept in a colony environment during early adulthood could already exhibit circadian rhythms in locomotion at the age of 2 days (Eban-Rothschild et al., 2012; Beer et al., 2016; Fuchikawa et al., 2016). Recently, it has been shown that temperature plays a pivotal role in the ontogeny of circadian activity rhythms as well. Bees kept at 35°C, which corresponds to the core hive temperature, for the first 24 h of their life display rhythms already at the age of 1–2 days while the bees kept at typical laboratory temperatures of 25°C need a few days longer (Giannoni-Guzman, 2016; Nagari et al., 2017). This is not surprising, because the correct temperature is important for a normal brain development and affects behavioral performance in the adult bee (Tautz et al., 2003; Groh et al., 2004; Jones et al., 2005). The honey bee brain further matures during adulthood, which is indicated by an age-dependent plasticity in mushroom bodies (Farris et al., 2001; Groh and Meinertzhagen, 2010). In our experiments (performed at 30°C), all honeybees needed more than 2 days until they exhibited circadian rhythms in locomotion, with or without social contact to a mini-colony. This ontogeny of circadian rhythms is slightly later than reported in the above mentioned studies, that showed circadian ontogeny in young honeybees at the age of 2 days or earlier, when kept under social contact or hive temperature (Eban-Rothschild et al., 2012; Beer et al., 2016; Fuchikawa et al., 2016; Giannoni-Guzman, 2016; Nagari et al., 2017). Nevertheless, our results comply with other behavioral studies indicating that the circadian system is not fully matured in newly emerged honey bees (Moore et al., 1998; Toma et al., 2000; Bloch et al., 2001; Eban-Rothschild et al., 2012; Beer et al., 2016; Giannoni-Guzman, 2016). Maturation speed in young honey bees thereby seems to depend on at least two factors: temperature and colony environment (possibly indirectly because of colony thermoregulation).

The solitary mason bee on the other hand shows circadian rhythms in locomotion right after emergence, which led us to the conclusion that the circadian system of this bee is fully matured at emergence. In contrast to the honey bee, temperature seemed to have no effect on the ontogeny of rhythmicity in solitary bees and most bees were rhythmic in both temperature treatments, which adds to our interpretation that development is largely finished at the time point of emergence of solitary bees compared to honey bees. Similarly, newly emerged fruit flies and tsetse flies, as examples of other holometabolic insects, also showed circadian behavior in locomotion (Brady, 1988; Sehgal et al., 1992).

### PDF Neurons of the Honey Bee Clock Are Not Fully Developed at the Time of Emergence

We found that the number of neurons expressing PDF increased moderately but significantly after honey bee emergence, which supports our hypothesis of an immature circadian clock in newly emerged honey bees. Similarly, Bloch et al. (2003) reported that the number of clock protein PERIOD (PER) expressing cells in the brain of foragers is higher than in the brain of nurses (Bloch et al., 2003). In spite of the fact that the molecular circadian clock ticks already in nurses (Fuchikawa et al., 2017), this early clock appears immature. For example, *per* mRNA levels are higher in forager brains than in nurses and show circadian cycling only in foragers, while in nurse bee brains, per transcript levels are not rhythmically produced (Toma et al., 2000; Bloch et al., 2004). The lack of cycling or cycling with a diminished amplitude of clock gene transcripts in nurse bees was later proven to be task related and consistent with the lack of rhythmic behavior in nursing bees (Shemesh et al., 2007, 2010). The growing clock network during aging of the adult honey bee, as we have observed it in our study, may promote the integration of new functions into the circadian system. It may be that the growing network also gains complexity during aging. Our recent analysis of the PDF network in nurses and foragers demonstrated that peptide levels oscillated with slightly different phases in different regions of forager brains, while they were highly synchronous in the brains of nurse bees (Beer et al., 2018). This indicates a higher level of complexity in the transfer of time-related information to brain areas with different functions in forager bees, which may relate to the complex tasks of foragers outside the hive. Interestingly, the number of positively stained PDF cell bodies stayed always high throughout the day in forager bees and was highest at the end of the subjective night/beginning of the subjective day in nurse bees (Beer et al., 2018). Therefore, we chose the subjective morning for quantification of the cell number in the differently aged bees in the here presented study. It is not clear, if the difference in cycling of PDF levels in different brain areas depends on developmental stage or task, but the maturation of the circadian clock seems to correlate with the age dependent polyethism in the honey bee. Due to the time dependent PDF peptide levels in PDF neuron arborizations observed in our recent study (Beer et al., 2018), we limited our analysis to counting PDF cell bodies, which was more consistent throughout different ages/tasks of the honey bee (at least for day times with high expression levels). Future studies, under controlled laboratory conditions and in the social hive environment, may dissect, which components of the honey bee clock are subjected to age- or task-related plasticity. Furthermore, the temperature dependency of clock system maturation in adult bees may be investigated also on the neuroanatomical level in the future.

### Emergence With an Immature Circadian Clock as Trait of Social Life Style in the Honey Bee

Sociality has evolved in multiple groups, such as mammals (e.g., mole rats), insects (e.g., some bees, some wasps and ants) or even microorganisms, because of evolutionary benefits from group hunting, development of resistance against disease or predators and increased reproductive success (Alexander, 1974; Crespi, 2001). In bees, eusociality (full sociality) was found only in few bee species (e.g., *Apis* and *Melipona* species), but social behavior has been found in several other bee species in different nuances (e.g., primitively social bumble bees or facultatively social sweat bees) (Shell and Rehan, 2018). In case of the honey bee, improved colony defense is one clear benefit of sociality, but also the vast amount of offspring production in a colony (Breed et al., 1990).

We propose in our research another adaptation that accelerates colony growth: a differentially timed development in social and solitary bees that results in the emergence of social bees with an immature circadian system. This may provide an evolutionary advantage on both colony and individual level. Even with an immature circadian system, honey bees can survive being under the protection of a social community and they can undertake in-hive tasks in the colony. A mismatch of activity to daytime is not relevant for avoiding predators or foraging success, because the young honey bee is provisioned by the older foragers. An immature circadian clock may be even beneficial to the young honey bee, which takes care of the brood day and night, because honey bees have so far shown a lack of ill effects caused by arrhythmic behavior, while other animals are strongly affected (Bloch et al., 2013).

As we expected, the number of PDF neurons does not change during aging of the solitary bee in our study. This fits, like the activity rhythms in young solitary bees, to our hypothesis of an already fully matured circadian system in the newly emerged solitary mason bee. Similarly, Withers and co-authors found that *A. mellifera* showed continued brain development during aging, while *Osmia lignaria* did not (Withers et al., 2008). Proportions of mushroom bodies compared to Kenyon cell bodies in newly emerged *Osmia* were just as high as in foraging honey bees. Furthermore, the proportions did not change during aging in *Osmia*, while this was the case in *Apis* (Withers et al., 2008). Our findings point to a shift of emergence to an earlier developmental stage of the circadian system in the social honey bee as compared to the solitary bee. It is certainly true, that there are other differences in the life style of *A. mellifera* and *O. bicornis* and we want to state clearly that a causal association of sociality and difference in clock development cannot be drawn from our results. For example, *O. bicornis* is a univoltine species, but *A. mellifera* produces several generations per year (Raw, 1972). Nevertheless, sociality appears to be the most striking difference in life style of these bees and other adaptations may have co-evolved with the specific life style, like for example the almost continuous reproduction in honey bee colonies.

We limited our investigation of clock development in bees to one social and one solitary species out of technical reasons. Especially the clock in solitary bee species is so far extremely little investigated and future studies may show, whether our findings can be generalized to other social and solitary bees. There are a few studies on circadian plasticity in other social hymenoptera. In bumblebees, which display primitive sociality, circadian plasticity in activity and number of PDF expressing neurons has been associated with body size and task rather than age (Yerushalmi et al., 2006; Weiss et al., 2009). For ants, a similar dependency of circadian rhythms on task, like in the honey bee, has been postulated: forager ants display rhythmic behavior and cycling levels of clock gene transcripts, which was not the case in nurse ants (Ingram et al., 2009; Fujioka et al., 2017). In addition, age-dependent modulations of the circadian clock have been reported in ants. Ingram and coauthors (Ingram et al., 2009) determined an increased level of overall per transcript in old foragers of the harvester ant species *Pogonomyrmex occidentalis*, which is reminiscent of the situation found in *A. mellifera* (Toma et al., 2000; Bloch et al., 2004; Abreu et al., 2018). The modulation of circadian behavior via contact to other colony members seems to play a role in rhythmic behavior of ants as well, because young ants in contact with old ants showed only weak behavioral rhythms (Fujioka et al., 2019). Interestingly, the clocks of different mammals, including humans, showed progressed maturation after birth like the honey bee (Rivkees, 2003; Weinert, 2005). This raises the question, whether social life style (in particular the protection of the colony) is a prerequisite for evolution of emergence with an immature circadian system, or the other way around or these traits rather co-evolved in honey bees (and possibly other eusocial hymenoptera like ants). Further studies are needed to dissect the evolutionary background of maturation processes of the circadian system in different animals.

Summarized, our comparative study demonstrates a differentially timed clock development of the social honey bee and the solitary mason bee. While the circadian system of the mason bee seems completely matured at emergence, the honey bee clock shows continued maturation after emergence. The early emergence with an immature circadian system benefits the colony community of the social bee and this adaptation of the honey bee clock may have evolved along with this species’ social life style.

## Data Availability Statement

The raw data supporting the conclusions of this article will be made available by the authors, without undue reservation.

## Author Contributions

KB collected and analyzed the data and wrote the manuscript. CH-F and KB conceived the project. CH-F contributed funding and revised the manuscript. Both authors contributed to the article and approved the submitted version.

## Conflict of Interest

The authors declare that the research was conducted in the absence of any commercial or financial relationships that could be construed as a potential conflict of interest.
